# Imidazolized Activated Carbon Anchoring Phosphotungstic Acid as a Recyclable Catalyst for Oxidation of Alcohols With Aqueous Hydrogen Peroxide

**DOI:** 10.3389/fchem.2022.925622

**Published:** 2022-06-29

**Authors:** Min Zheng, Huiting He, Xiangzhou Li, Dulin Yin

**Affiliations:** ^1^ College of Material Science and Engineering, Central South University of Forestry and Technology, Changsha, China; ^2^ College of Physics and Chemistry, Hunan First Normal University, Changsha, China; ^3^ National & Local Joint Engineering Laboratory for New Petro-chemical Materials and Fine Utilization of Resources, College of Chemistry and Chemical Engineering, Hunan Normal University, Changsha, China

**Keywords:** phosphotungstic acid, 2-methylimidazole, activated carbon, oxidation, benzyl alcohol

## Abstract

Keggin-type phosphotungstic acid (HPW) supported on imidazolyl-activated carbon (AC-COIMI-HPW) catalysts was prepared, which was used to catalyze the oxidation of benzyl alcohol with aqueous H_2_O_2_. In the presence of AC-COIMI-HPW, the benzyl alcohol conversion of 90.2% with 91.8% selectivity of benzaldehyde was obtained at 90°C for 6 h in an acetonitrile aqueous solution. The catalyst exhibited an outstanding performance for the oxidation of various benzyl alcohols and aliphatic alcohols. In addition, the catalyst could be easily recovered and reused five times without significant deactivation. The characterization results showed that HPW was chemically bonded on the surface of the carbon material through an ionic bond. It is proposed that the combination of the imidazole cation with the HPW anion could not only tune the redox catalytic properties of the PW anion but also enhance the compatibility of the catalyst in the reaction medium, thereby improving the catalytic performance.

## 1 Introduction

The oxidation of alcohols to aldehydes and ketones plays an important role in the synthesis of organic compounds such as agrochemicals, pharmaceuticals, and dyestuff in the industry and laboratory (Mandal et al., 2013). In the traditional chemical oxidation process, several common oxidants such as dichromate or permanganate were usually used and exhibited strong oxidative properties. However, these oxidants are often associated with poor selectivity, high toxicity, and harmful by-products of heavy metals, which result in serious environmental pollution, increasing the difficulty and cost of subsequent treatment. Aerobic oxidation is an attractive strategy for the oxidation of alcohols. Ten years ago, ruthenium hydroxide supported on silica was used as an efficient catalyst for the aerobic oxidation of monoterpene alcohols (Costa et al., 2011). Recently, layered double hydroxide-supported Cu^0^ nanoparticles (Choudhary et al., 2019), Au nanoparticles (Karimi et al., 2020), and polymeric ionic liquid microspheres/Pd nanoparticles (Wu et al., 2020) have been designed for the oxidation of alcohols. It was presented that primary and secondary alcohols can be efficiently oxidized using CoFe_2_O_4_@HT@Imine-Cu II and TEMPO in the air atmosphere (Salimi et al., 2020). However, the above-mentioned catalysts were expensive and generally require a longer reaction time at high temperatures to achieve good catalytic performance. Therefore, it is still a challenge to develop an oxidation system that is easily available, stable, inexpensive, environmentally acceptable, and promotes selective oxidation under mild reaction conditions.

The green chemical industry is aimed at reducing or eliminating the use and generation of hazardous substances. Hydrogen peroxide (H_2_O_2_) has been recognized as a green oxidant because of it being environmentally friendly, inexpensive, and easy to handle (Shi et al., 2021). In the past period, much research has been devoted to the selective oxidation of alcohols in the presence of H_2_O_2_. Tungstate ions supported on imidazolium framework (Hosseini et al., 2017) or magnetic mesoporous silica (Norouzi et al., 2019) and molybdate ions immobilized on ionic liquid–modified CMK-3 (Hosseini-Eshbala et al., 2020) were employed for the oxidation of benzyl alcohols. It is noteworthy that iron chloride ionic liquid immobilized on SBA-15 (Cang et al., 2015) and cheap iron (III) tosylate (Zhao et al., 2018) was applied for selective oxidation of alcohols, whereas a large amount of H_2_O_2_ was consumed in the presence of iron ion. Chromium borophosphate synthesized by the solution combustion method at 800°C was also tested for the oxidation of benzyl alcohol (Demet Ozer, 2020).

Polyoxometalates (POMs) are a category of transition metal oxide clusters with hollow structures and relatively large surface areas (Hao et al., 2018). Benefiting from the modifiable redox and acidic properties, POMs have received widespread attention in the catalytic oxidation of alcohols with H_2_O_2_. However, the direct use of pure POMs has some problems such as low activity and difficulty in recovering the catalyst due to its water solubility. In order to solve this problem in the oxidation of alcohols with H_2_O_2_, a lot of research has been devoted to fabricating insoluble POM catalytic composite materials, including macromolecular-like polyoxometalate-based ionic hybrid with a polyamine (Chen et al., 2014; Su et al., 2014), dendritic phosphotungstate structure (Chen et al., 2014; Sadjadi et al., 2019), squeezing POMs in ionic liquids (Li et al., 2015; Keshavarz et al., 2019), and immobilizing onto mesoporous molecular sieves (Tan et al., 2012; Dong et al., 2014). In order to promote selective oxidation of alcohols, some carbon materials such as multi-wall carbon nanotubes modified with ionic liquids (Hajian et al., 2017: Liu et al,2014) and ionic liquid–functionalized graphene oxide (Liu et al., 2013; Zheng et al., 2019) were also used as a support for POMs. It should be observed that there exist alkaline sites or ion exchangeable sites on supports that are used for immobilization of POMs.

Activated carbon (AC) is a large-tonnage industrial carbonaceous material, which might be produced from biomass formed by natural photosynthesis. AC has a high specific surface area, pore structure, and surface functional groups (Alslaibi et al., 2014). Therefore, it has a wide range of applications in the fields of adsorption of heavy metals (Koohzad et al., 2019), supercapacitor electrodes (Fan et al., 2011), decolorization (Shahbazi et al., 2020; Liu et al., 2021), and catalyst supports (Yao et al., 2020; Xu et al., 2021; Costa et al., 2021). However, it is generally believed that the most attractive feature of activated carbon materials is their specific and controllable surface reactivity. Usually, the oxidation of activated carbon with nitric acid can increase carboxylic acid sites (Ternero-Hidalgo et al., 2016) or convert to basic sites by NaOH treatment (Cazettaa et al., 2011). Furthermore, imidazolyl-activated carbon has been recently prepared by ethylenediamine treatment (Liu et al., 2019). Based on the aforementioned facts, phosphotungstate catalytic active sites were constructed by chemically modifying the surface carboxylation and imidazolization of carbonic materials with 2-methylimidazole, and their catalytic performances in the oxidation of alcohols were investigated.

## 2 Experiment

### 2.1 Materials

Phosphotungstic acid was purchased from Aladdin. Benzyl alcohol was purchased from Xilong Science Co., Ltd., and 30% hydrogen peroxide solution was provided by Baling Branch, SINOPEC. Other reagents were purchased from Sinopharm Chemical Reagent Co., Ltd. (Shanghai, China). All chemicals were not further purified prior to use.

### 2.2 Catalyst Preparation

#### 2.2.1 AC-COOH

**Scheme 1:**
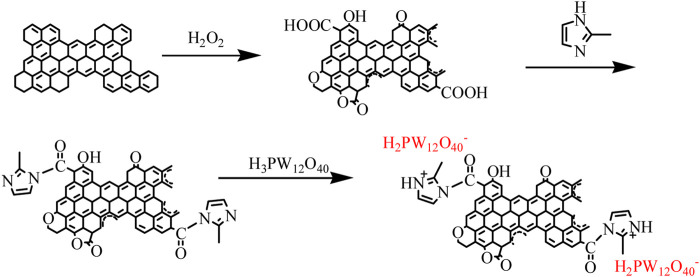
Preparation of AC-COIMI-HPW.

The preparation process of the catalyst is shown in Scheme 1. Unlike the usual carboxylation of carbon materials with hazardous nitric acid, a green oxidation method of the activated carbon surface was developed. Generally, 10 g of activated carbon was added to a three-necked flask equipped with a condenser and a thermometer, 50 ml of 50% hydrogen peroxide and 5 ml of 12 mol L^−1^ H_2_SO_4_ aqueous solution were added, and then stirred at 80°C in an oil bath for 2 h, while which another 25 ml of 50% hydrogen peroxide was added drop by drop for 1 h. When the oxidative treatment was completed, the reaction mixture was then filtered and washed repeatedly with deionized water until no SO_4_^2−^ ion could be detected in the barium chloride solution. The resulting solid was dried in an oven at 120°C for 6 h. The oxidized activated carbon was designated as AC-COOH.

#### 2.2.2 AC-COIMI

Direct imidazolization of oxidized activated carbon was carried out. Generally, 50 g of 2-methylimidazole was first added in a three-necked flask; then, 10 g of AC-COOH was added, equipped with a condenser and connected to an anhydrous calcium chloride tube, a thermometer, and a silicone oil bath; the temperature was increased to 180°C at 3°C min^−1^ and maintained for 10 h. After the operation of amidation, the oil bath was removed, the flask was cooled to room temperature, and 70 ml of acetone was added. Once sealed overnight, it was filtered, and extraction of the residue was launched with acetone for 10 h until 2-methylimidazole did not exist in the Soxhlet apparatus monitored by a UV-vis spectrometer and dried in a vacuum to collect the acyl-imidazolized activated carbon AC-COIMI.

#### 2.2.3 AC-COIMI-HPW

First, a certain amount of HPW was dissolved in ultrapure water to obtain a series of standard solution. Then, 1.00 g of AC-COIMI was added to the corresponding solution, standing for 24 h. The parent liquor was separated by centrifugation, washed with ultrapure water until no phosphotungstic acid exists in the liquid monitored by using a UV-vis spectrometer, and dried in vacuo overnight. The resultant material is designed as an AC-COIMI-HPW catalyst.

### 2.3 Adsorption Isotherm Determination

Accurately weighed AC-COIMI samples were put in a series solution in conical bottles with different concentrations of HPW and then fixed in a constant temperature shaker, shaken at 25°C for 5 h or more to adsorption equilibrium. Once equilibration was attained, the centrifuged mother liquor was taken out, and the concentration of HPW in the liquor was detected by UV-vis spectrophotometry from the absorbance at 258 nm. The adsorption quantity of HPW on an AC-COIMI catalyst was calculated by measuring the concentration difference of the initial and last solution.

### 2.4 Catalyst Characterizations

The Fourier transform infrared spectra (FT-IR) of the samples were collected by the KBr pellet technique on a Nicolet 370 infrared spectrophotometer in the range of 400–4,000 cm^−1^. The X-ray diffraction (XRD) patterns of the sample were recorded by using a Bruker diffractometer with Cu Kα radiation and diffraction angle (2θ) ranging from 10° to 80°. A DXR laser Raman microscope (laser wavelength 780 nm) was used; the wavelength range was 50–3,250 cm^−1^, the exposure time was 5 s, the number of exposures was 10, and the laser intensity was 5 mW. The Thermogravimetric and derivative thermogravimetric (TG-DTG) experimental results were obtained on a Netzsch Model STA 409PC instrument, from room temperature to 800°C at 10°C/min using α-Al_2_O_3_ as the standard material. The UV-Vis spectrum (190∼900 nm) was measured by using a UV-2450 apparatus of Shimadzu. Chemical analysis of W in the reaction was carried out by inductively coupled plasma-emission spectrometry (ICAP 7200, Thermo Fisher Scientific Co., Ltd., United States)

### 2.5 Catalytic Reaction Tests

Benzyl alcohol and a certain amount of catalyst were added into a 25-ml round bottom flask. Then, a certain amount of solvent was added, and the mixture was heated to the specified temperature. When the temperature was stable, a quantitative 30 wt% H_2_O_2_ was added, and timing was started. After the reaction was completed, the mixture was cooled to room temperature and centrifuged. Then, 100 μl of the reaction solution was sucked into the PV tube and diluted 10 times before passing through the ultrafiltration membrane. The conversion and selectivity were determined by GC using an internal standard method (Nexis GC-2030). In reuse experiments, the deposited catalyst was washed with the solvent three times for the next run.

## 3 Results and Discussion

### 3.1 Catalyst Characterization

#### 3.1.1 FTIR

**FIGURE 1:**
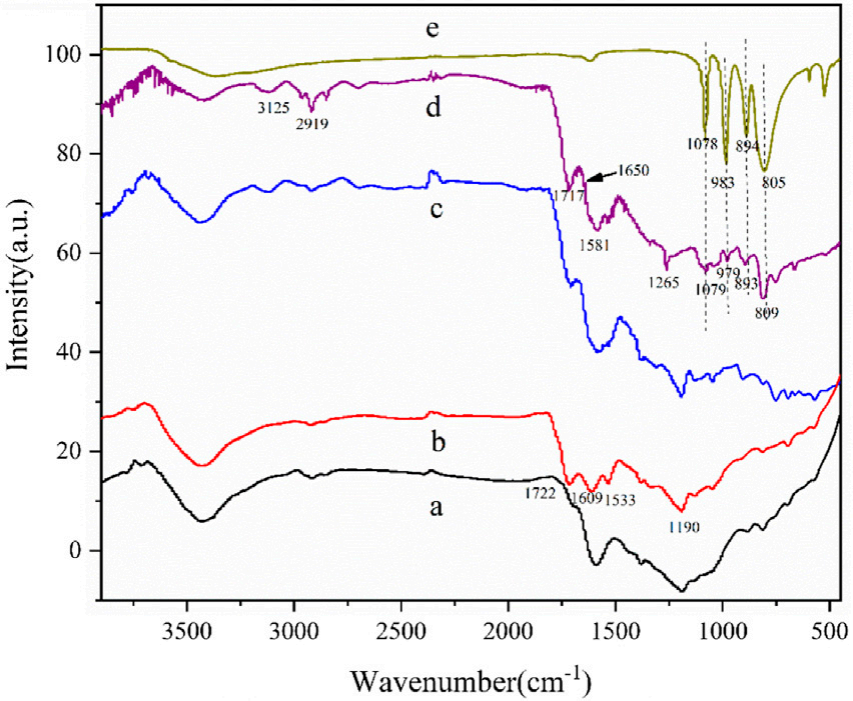
FT-IR spectra of (a) AC, (b) AC-COOH, (c) AC-COIMI, (d) AC-COIMI-HPW and (e) HPW.

It is shown, in Figure 1, that the carbonyl stretching vibration peak at 1722 cm^−1^ from the oxidized activated carbon can be seen as higher than the original activated carbon without oxidation. In addition, the absorption peaks at 1,609 and 1,533 cm^−1^ belong to the C=C stretching vibration zone of the fused ring aromatic hydrocarbon skeleton on the activated carbon. The peak at 1,190 cm^−1^ is attributed to the stretching vibration of the carboxyl group, lactone group, or the phenolic hydroxyl group on the activated carbon. This result proves the existence of abundant functional groups on activated carbon, which also provides basic conditions for us to modify activated carbon by chemical methods. As compared to AC-COIMI, AC-COIMI-HPW retains its 2-methylimidazole structure and therefore presents the characteristic peak of the imidazole skeleton. The peak at 3,125 cm^−1^ belongs to the C-H stretching vibration on the unsaturated carbon of fused ring aromatic hydrocarbons on imidazole or activated carbon. The peak at 2,919 cm^−1^ belongs to the C-H stretching vibration of the saturated carbon of the methyl group on 2-methylimidazole. The peak at 1,717 cm^−1^ belongs to the stretching vibration of the carbonyl group. Here, 1,650 cm^−1^ represents the -C=N stretching vibration on the imidazole skeleton, and 1,265 cm^−1^ is attributed to the C-N bending vibration peaks on the imidazole ring. The four main characteristic bands of pure phosphotungstic acid are located at 1,078 (P-O_a_), 983 (W=O_d_), 894 (W-O_b_-W with co-angular octahedron), and 805 cm^−1^ (W-O_c_-W with co-lateral octahedral), and the corresponding peaks can be found in AC-COIMI-HPW. The characteristic peak of heteropoly acid has a slight deviation, which is mainly because the lone pair of electrons in the organic cation extends into the inorganic framework of the heteropoly anion, which makes the formation of a strong electronic force between the two so that the characteristics of both sides and the absorption peaks have shifted to a certain extent, and these shifts and changes indicate the interaction between the phosphotungstate anion and imidazolium cation (Mohamadi et al., 2020). The aforementioned results showed that the modification of activated carbon is successful. No obvious Keggin structure’s absorption peak is observed in the catalyst, which confirms that the phosphotungstic acid species is highly dispersed, which is consistent with the following XRD results.

#### 3.1.2 Raman Spectra

**FIGURE 2:**
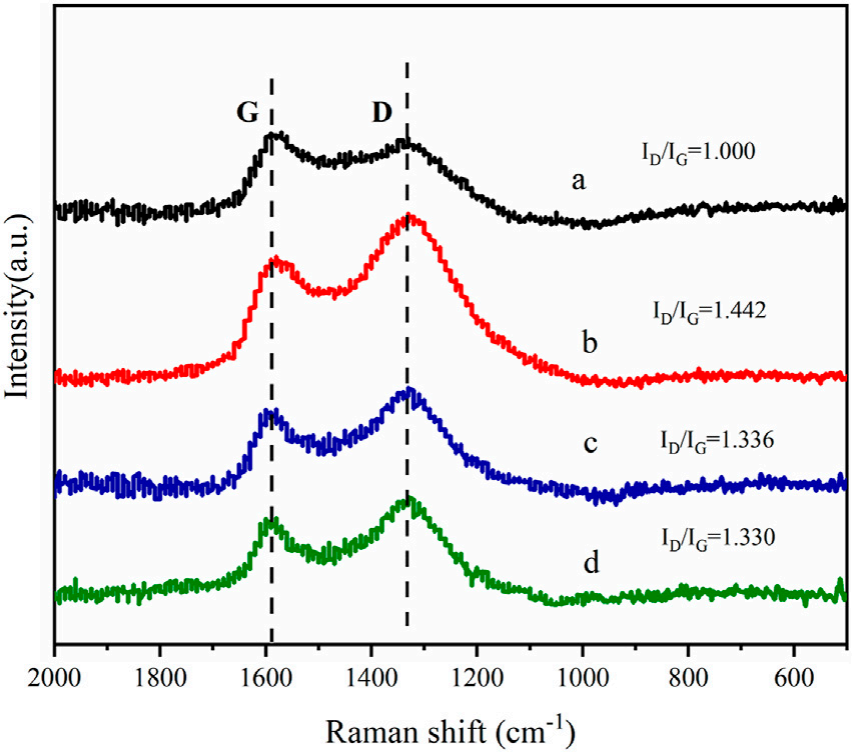
Raman of (a) AC, (b) AC-COOH, (c) AC-COIMI and (d) AC-COIMI-HPW.

The Raman spectra of AC-COIMI-HPW and its precursors AC, AC-COOH, and AC-COIMI are presented in Figure 2, and the changes in the carbon skeleton of the activated carbon after each step of modification are presented. These carbon materials mainly have two broad peaks at 1,580 and 1,336 cm^−1^. The peak at 1,580 cm^−1^ is the G peak, which represents the graphite-like structure of the central sp^2^ carbon skeleton. Another peak at 1,336 cm^−1^ is D which represents the non-graphitized amorphous structure in the material (Shimodaira et al., 2002). The intensity ratio I_D_/I_G_ of peak D and peak G can represent the relative content of the graphene-like structure of the carbon material. Analyzing the I_D_/I_G_ value obtained by Gaussian fitting, the I_D_/I_G_ of the activated carbon after the nitric acid treatment becomes larger, indicating that the non-graphitized amorphous degree of the activated carbon is increased by oxidation treatment because the graphene-like fused-ring aromatic structure at each layer is destroyed to a certain extent. After the subsequent modification of 2-methylimidazole, it seems the degree of graphitization increased again. The main reason may be that the G peak represents C (sp^2^), and the D peak represents the vibration of C (sp^3^). The introduction of imidazole makes the sp^2^ type carbon have a certain degree of increase so that the degree of G peak has a certain increase. The subsequent phosphotungstic acid modification shows that it has little effect on the Raman spectra of AC-COIMI because HPW cannot change the ratio of both sp^2^ and sp^3^ carbon in the carbon material.

#### 3.1.3 XRD

**FIGURE 3:**
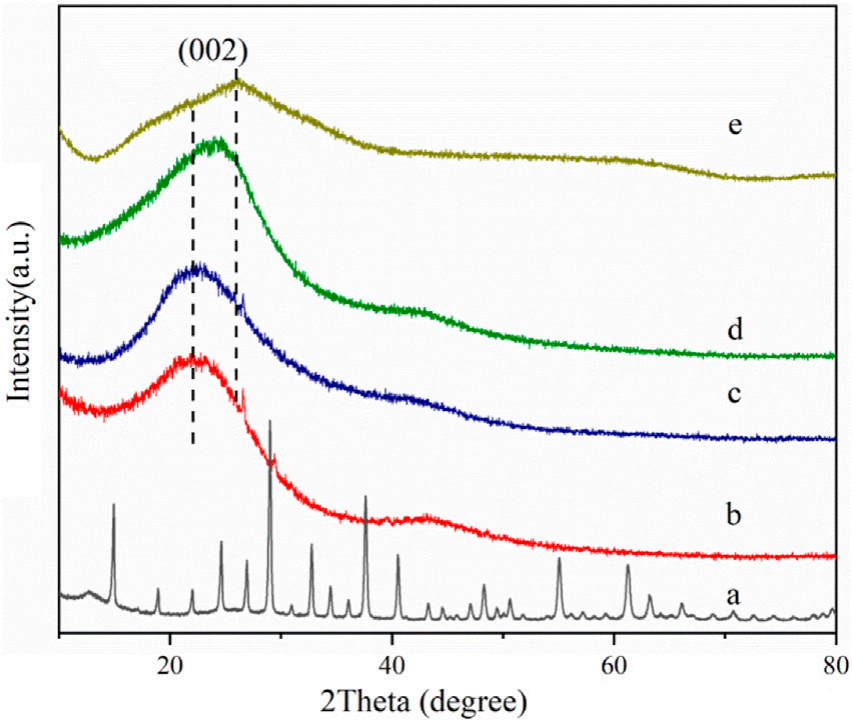
XRD patterns of (a) HPW, (b) AC, (c) AC-COOH, (d) AC-COIMI and (e) AC-COIMI-HPW.

Figure 3 shows the wide-angle X-ray diffraction curves of phosphotungstic acid, oxidized activated carbon, and phosphotungstic acid-loaded activated carbon. It shows the characteristic diffraction peaks of phosphotungstic acid crystals. The Keggin cubic structure diffraction peaks of HPW mainly appear at 10.3°, 18.0°, 20.6°, 23.1°, 25.2°, 29.3°, 34.6°, and 37.8°, which are consistent with the literature reports (JCPDS-#7521–25) (Atia et al., 2008; Costa et al., 2021). The diffraction curve of the oxidized activated carbon showed the dispersion peak of the amorphous phase. In the case of AC-COIMI-HPW, it was observed that it does not show an obvious phosphotungstic acid diffraction peak, and the 2θ value of the diffraction peak representing the (002) crystal plane of the graphite structure has shifted from 21.9° to 25.7°. From Bragg Eq. 2 d_hkl_ sin*θ* = *n λ*, it seems to indicate that the graphene-like structure in AC-COIMIHPW is compressed, and the particle size is smaller by the reaction of AC-COIMI with HPW. This may be because the subsequent modification operations have changed the degree of graphitization of carbon materials. The results are good and agree with the FTIR result, and it shows that the phosphotungstate species are highly self-dispersed on the activated carbon from the adsorption process, in other words, which also means that HPW is loaded uniformly on the imidazole site in the form of a single molecule.

#### 3.1.4 TG-DTG

**FIGURE 4:**
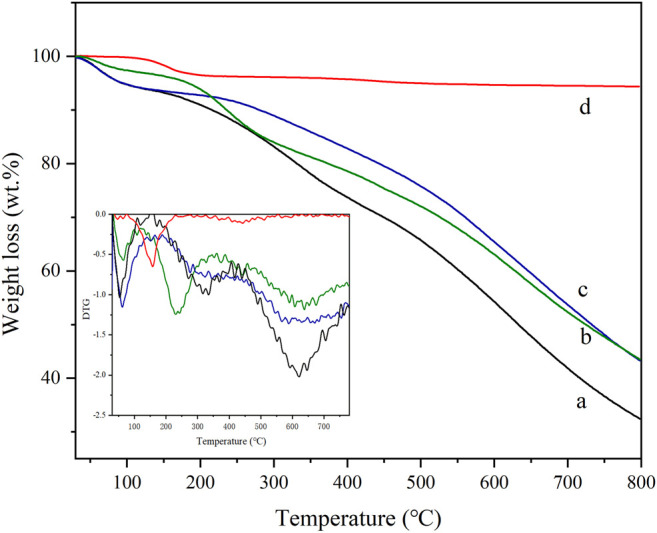
TG and DTG of (a) AC-COOH, (b) AC-COIMI, (c) AC-COIMI-HPW and (d) HPW.

The TG-DTG profile of four samples is shown in Figure 4. The weight loss curve of HPW in Figure 4d indicates that the first weight loss peak between 0°C and 100°C is the removal of phosphotungstic acid physical adsorption water in three carbon materials. The structural water of phosphotungstic acid forms [H_2_O. . .H^+^. . .OH_2_] ions by hydrogen bonding with acidic protons, and its water loss peak is located at 100°C–200°C, and only pure HPW loses weight at about 200°C. However, it is mainly due to a small amount of weight loss of the decomposition of phosphotungstic acid between 300–600°C, that is, the Keggin structure is destroyed (Tan et al., 2012). The total weight loss of HPW was ca. 5.5% ranging from 600°C to 800°C. In the weight loss curve of AC-COOH shown in Figure 4a, the weight loss peak of the adsorbed water on the surface of the carbon material below 100°C was observed. In addition, the weight loss (about 15%) between 150°C and 250°C is mainly the decomposition of the carboxyl group on the carbon material, which further verified the existence of the abundant carboxyl group on the oxidized modified carbon material. The TG curve of AC-COIMI-HPW is shown in Figure 4b. The first weight loss can be assigned to the physical adsorption of water. The second stage ranging from 150°C to 450°C corresponds to the decomposition of the oxygen-containing groups on the surface of the carbon material and the decomposition of the imidazole organic skeleton. The weight loss during this stage is about 38.3%. In the third stage up to 800°C, the weight loss can be considered as the decomposition of the residual oxygen-containing groups in the activated carbon. Figure 4c shows the weight loss curve of AC-COIMI-HPW, and the weight loss peak in the first stage is still the physical adsorption water lost by the catalyst. The corresponding weight loss can be found in the AC-COIMI ranging from 150°C to 450°C, it includes the decomposition of oxygen-containing groups and imidazole skeletons and water of the phosphotungstic acid structure. The increase in the decomposition temperature of bound water in phosphotungstic acid is also proof of our successful combination of imidazole and phosphotungstic acid, improving the stability of phosphotungstic acid on the activated carbon.

#### 3.1.5 BET

**FIGURE 5:**
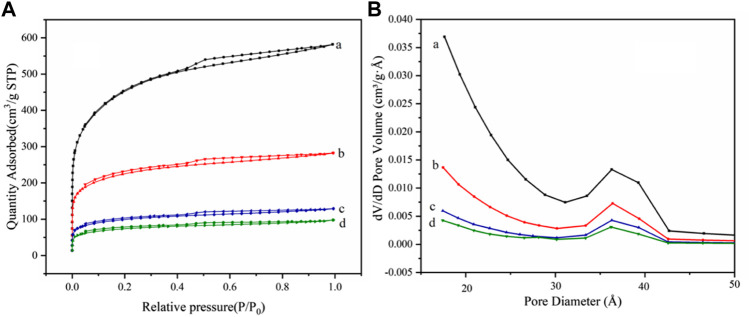
**(A)** N_2_ adsorption–desorption isotherms and **(B)** pore size distribution for (a) AC, (b) AC-COOH, (c) AC-COIMI and (d) AC-COIMI-HPW.

The specific surface areas of AC, AC-COOH, AC-COIMI, and AC-COIMI-HPW are determined using the BET method, and the isotherms of nitrogen absorption and desorption are shown in Figure 5A. It can be seen that N_2_ adsorption isotherms of four samples showed type IV adsorption isotherms, indicating the presence of the mesoporous structure (Park et al., 2014; Liu et al., 2019).

The pore size distribution of the sample is shown in Figure 5B. It was found that the pore size distribution of AC-COOH, AC-COIMI, and AC-COIMI-HPW was decreased obviously, indicating the mesoporous structure may be blocked or destroyed during the oxidation and subsequent modification process.

From Table 1, the specific surface area and pore volume of the carbon materials are gradually decreased with oxidation, imidazolization, and binding HPW. It was shown that the texture of the carbon materials has a certain degree of change after a series of chemical modifications. It could be found that the surface carboxyl density of the activated carbon was vastly increased after the oxidation. The imidazole group density (N-Base) is not equivalently matching with the carboxyl amount; this may be the existence of the decarboxylation of oxidized carbon during the amidation of the carboxyl imidazole salt in the alkaline 2-methylimidazole medium.

**TABLE 1:** Surface area and pore properties of four carbonic material samples.

Sample	BET surface area (m^2^/g)	Pore volume (cm^3^/g)	Density (μmol/g)
AC	1,072	0.65	10 (-COOH)
AC-OOH	615	0.37	152 (-COOH)
AC-COIMI	316	0.19	103 (N-Base)
AC-COIMI-HPW	235	0.15	—

### 3.2 Catalytic Performance

#### 3.2.1 Evaluation of the Catalysts

The catalytic performance of AC-COIMI and AC-COIMI-HPW was investigated in the benzyl alcohol oxidation reaction, and the results are shown in Table 2. It could be seen from Table 2 that the conversion of benzyl alcohol was less than 5% in the presence of no catalyst or AC-COIMI (Table 2, entries 1 and 2), indicating that self-oxidation with H_2_O_2_ is very difficult in the reaction conditions. It is noteworthy that the catalytic performance was improved by HPW supported on AC (Table 2, entries 3 and 4), which brought an increase of 12 percentage points in the conversion and more than twenty in benzaldehyde selectivity. When HPW was supported on AC-COIMI (entry 7), the catalytic performance was further improved. The conversion raised 12 percentage points, and the benzaldehyde selectivity slightly increased in comparison with AC-HPW.

**TABLE 2:** Effects of various catalysts on the oxidation reaction of benzyl alcohol.

Entry	Catalyst	HPW loading (mmol/g)	Conversion (%)	Selectivity^a^ (%)
1	None	—	4.7	98.5
2	AC-COIMI	—	2.0	99.2
3	HPW	0.075	58.6	70.1
4	AC-HPW	0.075	70.4	91.8
5	AC-COIMI-HPW (1)	0.031	43.9	97.3
6	AC-COIMI-HPW (2)	0.061	66.8	96.7
7	AC-COIMI-HPW (3)	0.075	82.4	96.0
8	AC-COIMI-HPW (4)	0.087	90.2	91.5
9	AC-COIMI-HPW (5)	0.090	91.3	90.4

Reaction conditions: 4 mmol alcohol, 16 mmol H_2_O_2_, 30 mg catalyst, 90°C, 6 h, and 15 ml solvent (CH_3_CN: H_2_O, 1:3).

a Selectivity for benzaldehyde.

As the loading of phosphotungstic acid increased, the conversion of benzyl alcohol gradually is increased (Table 2, entries 6–10) due to the increase in the catalytic sites. However, the selectivity of benzaldehyde is decreased on the contrary, which was caused by the consecutive oxidation of benzaldehyde to benzoic acid. It is very interesting that the conversion of benzyl alcohol on AC-COIMI-HPW (3) is higher than that of AC-COIMI and HPW (Table 2, entries 2 and 3), which means the catalytic properties of HPW adsorbed on AC-COIMI were modified by the interaction of the phosphotungstic acid molecule with the imidazole site through adsorption on the AC-COIMI surface.

#### 3.2.2 Influence of the Reaction Medium

The solvent has an important influence on the oxidation of benzyl alcohol to benzaldehyde when heteropoly acid was used as a catalyst because solvent molecules can occupy the lower energy LUMO in the heteropoly acid (Majid et al., 2007). The effect of the reaction medium on the catalytic performance of AC-COIMI-HPW (4) was investigated, and the results are shown in Table 3. The oxidation of benzyl alcohol with H_2_O_2_ occurred on the catalyst with no additional solvent, but the catalytic performance is not attractive (entry 1).

**TABLE 3:** Catalytic oxidation of benzyl alcohol in various solvents.

Entry	Solvent	Temp. (°C)	Conversion (%)	Selectivity (%)^a^
1	—	90	52.3	88.5
2	Acetone	60	6.8	98.5
3	Ethyl acetate	80	13.5	88.9
4	Toluene	100	40.4	90.8
5	H_2_O	90	75.0	92.0
6	CH_3_CN	90	8.6	97.7
7	CH_3_CN: H_2_O(3: 1)	90	13.8	98.6
8	CH_3_CN: H_2_O(1: 1)	90	42.2	97.6
9	CH_3_CN: H_2_O(1: 3)	90	90.2	91.5
10	CH_3_CN: H_2_O(0.5: 3.5)	90	88.5	90.5

Reaction conditions: 4 mmol alcohol, 16 mmol H_2_O_2_, 30 mg catalyst, 90°C, 6°h, and 15 ml solvent.

a Selectivity of benzaldehyde.

For aprotic polar organic solvents, such as acetone and acetonitrile were added, the catalytic activity was ineffective (Entries 2 and 6). On the contrary, a relatively high conversion for benzyl alcohol was obtained in the presence of ethyl acetate and toluene, respectively. However, the selectivity of benzaldehyde was decreased close to 90% (entries 3 and 4). An interesting phenomenon occurred when the mixtures of acetonitrile and pure water were used in the reaction medium, in which the activity and selectivity were both improved (Entries 7–10). By adjusting the ratio of the mixed solvent, an optimal conversion was obtained at a 1: 3 volume ratio of acetonitrile with water.

#### 3.2.3 Influence of Reaction Conditions

**FIGURE 6:**
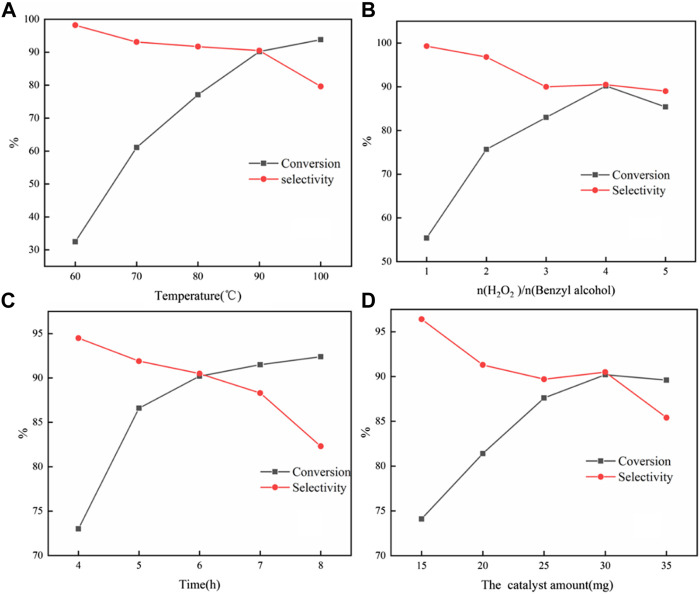
Influences of reaction conditions on the selective oxidation of benzyl alcohol with H_2_O_2_: temperature **(A)**; molar ratio of H_2_O_2_ to benzyl alcohol **(B)**; time; and **(C)** catalyst amount **(D)**. Reaction condition: 4 mmol benzyl alcohol, 16 mmol H_2_O_2_, 30 mg catalyst, 90°C, 6 h, and 15 ml solvent (CH_3_CN: H_2_O, 1: 3); for each figure, there is a specific parameter changed.

From Figure 6A, it was found that high selectivity could be obtained at 60°C; however, the conversion was very low. As the temperature further increased and the conversion increased, the selectivity declined sharply at 100°C. This might be due to high temperature conditions, which were more conducive to the conversion to the direction of benzoic acid. Therefore, 90°C was selected as the optimal reaction temperature.

It was found that the reaction conversion increases with the increase in the amount of H_2_O_2_ (Figure 6B). However, when the amount of H_2_O_2_ reached 5 eq., the conversion and selectivity dropped slightly. The reason for the slight decrease in the conversion rate could be understood as the increase in the concentration of hydrogen peroxide in the entire reaction system, which reduced the stability of hydrogen peroxide at this temperature, leading to an increase in self-decomposition. Therefore, the molar ratio of H_2_O_2_ to benzyl alcohol was four for the investigation of catalytic performance.

Figure 6C showed the effect of reaction time on the selective oxidation of benzyl alcohol. It can be found that the conversion could rise continuously with the extension of time. The selectivity could basically remain stable having a linear descent with the reaction time less than 7 h, after that sharp decline, which confirmed the consecutive oxidation of benzaldehyde to benzoic acid.

It could be seen from Figure 6D that the increase in the amount of catalyst could effectively increase the conversion. The increasing catalyst dosage can provide more catalytic sites. The collision probability of the substrate and the catalyst per unit time increased, so the oxidation reaction rate was effectively improved. At the same time, the generation of consecutive by-products would also be increased, so the selectivity of the catalyst decreased. With further increase to 35 mg, it speeded up the decomposition of hydrogen peroxide due to the excessive catalyst. The strong adsorption performance of the substrate prevents a part of the substrate from forming peroxide intermediates with phosphotungstic acid peroxide, which led to a decrease in conversion. This showed that an excess amount of the catalyst may be due to an inhibition effect during the reaction process.

#### 3.2.4 Substrate Adaptability

From the results of the substrate scope experiment with AC-COIMI-HPW in Table 4, it could be found that the regularity of the catalytic oxidation reaction of aromatic alcohols was that aromatic alcohols with electron-donating groups at the para position were more reactive than with electron-withdrawing groups. This may be attributed to the electron-donating group transferring electrons to the benzene ring, which increased the electron density of the benzene ring and activates the benzene ring, which made the aromatic alcohol more susceptible to oxidation. On the contrary, the presence of electron-withdrawing groups would reduce the conversion of benzyl alcohol. The activity of 2-bromobenzyl alcohol was further reduced compared with 4-bromobenzyl alcohol because of the existence of steric hindrance.

**TABLE 4:** Selective oxidation of various alcohols over the catalyst.

Entry	Substrate	Product	T (h)	Conversion (%)>	Selectivity^a^ (%)
1	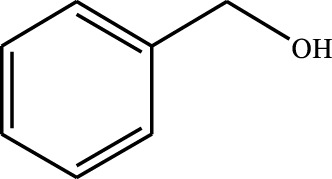	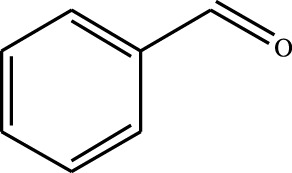	6	90.2	95.5
2	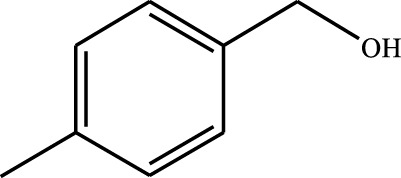	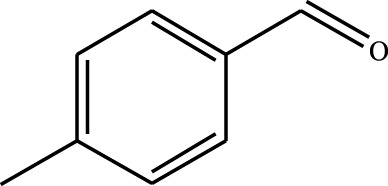	6	86.5	97.5
3	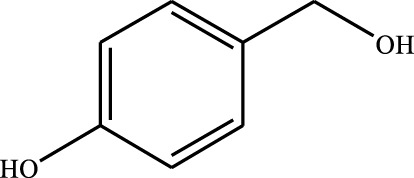	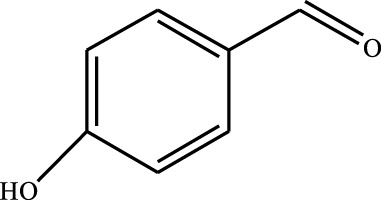	6	98.9	93.2
4	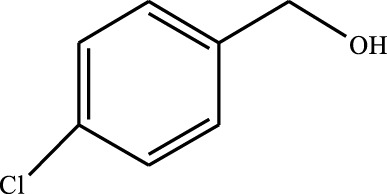	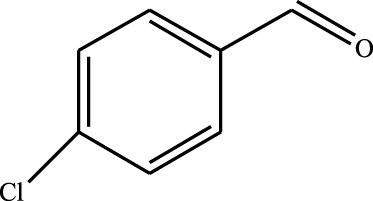	6	77.7	94.6
5	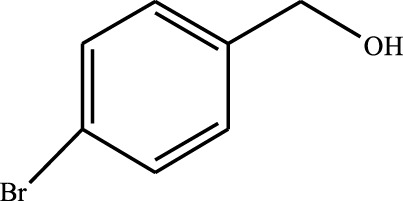	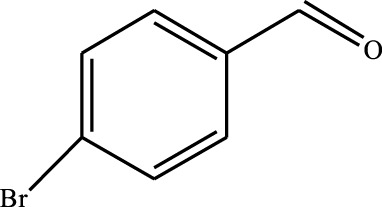	6	61.7	>99
6	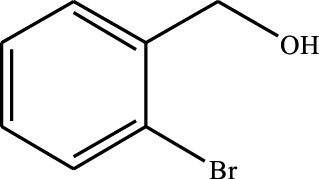	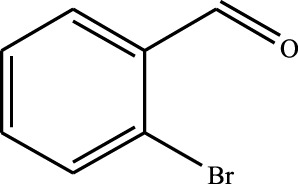	6	34.2	>99
7	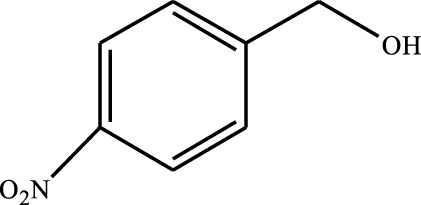	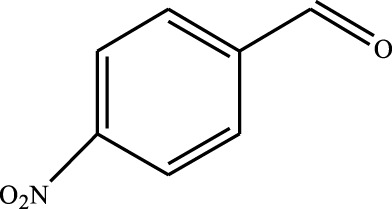	6	55.0	96.5
8	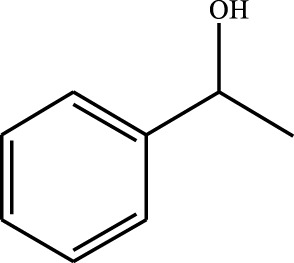	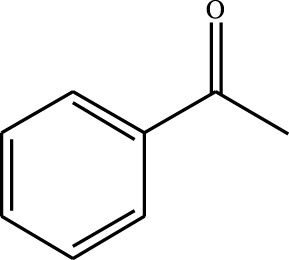	6	98.4	>99
9	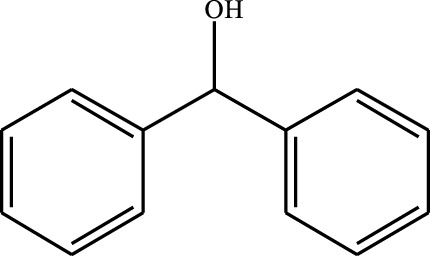	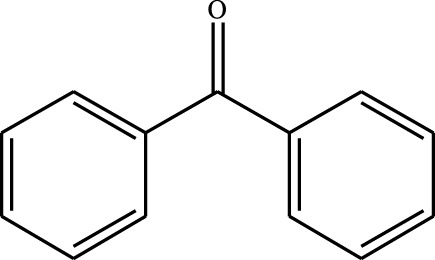	6	57.4	>99
10	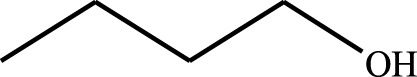	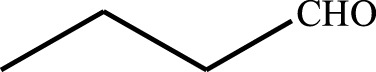	6	86.0	94.8
11	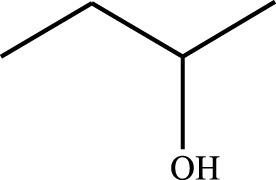	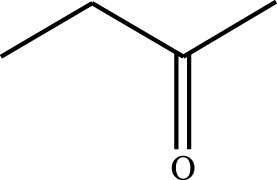	6	93.6	>99
12	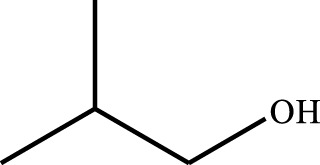	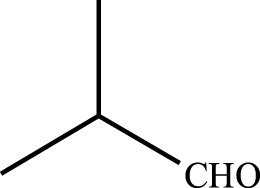	6	97.3	96.2
13	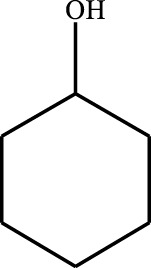	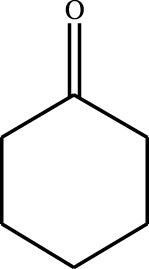	12	98.6	>99
14	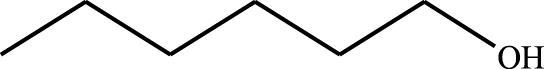	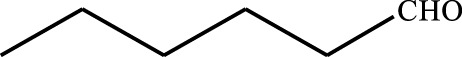	12	39.5	55.7

Reaction conditions: 4 mmol alcohol, 16 mmol H_2_O_2_, 30 mg catalyst, 90°C, 6 h, and 15 ml solvent (CH_3_CN: H_2_O, 1: 3).

a Selectivity of aldehydes or ketones.

The reason for the conversion rate of 1-phenylethanol better than benzyl alcohol might be the difference in bond energy values for the oxidation of C-H, the secondary C-H which in 1-phenylethanol is weaker than the primary C-H in benzyl alcohol (entries 1 and 8). This was confirmed by comparing the selective oxidation results of sec-butanol and n-butanol. Similarly, comparing cyclohexanol and n-hexanol, it was also found that the catalytic oxidation activity of cyclohexanol was much better than that of n-hexanol. Moreover, due to the large steric hindrance of the two benzene rings, the oxidized activity of benzhydrol was much low. Without exception, all secondary alcohols were metrologically oxidized to the corresponding ketone with H_2_O_2_; it was shown that the insertion of active oxygen species to the C–C bond in the ketone very difficultly occurred on the catalytic site of AC-COIMI-HPW; however, the active oxygen species on the catalytic site could generally insert to the C-H bond of the aldehyde group resulting in the formation of the corresponding acid. It is noted that modification is a very important strategy to inhibit or reduce the catalytic activity for the consecutive oxidation of the aldehyde group.

### 3.3 Catalytic Reusability

**FIGURE 7:**
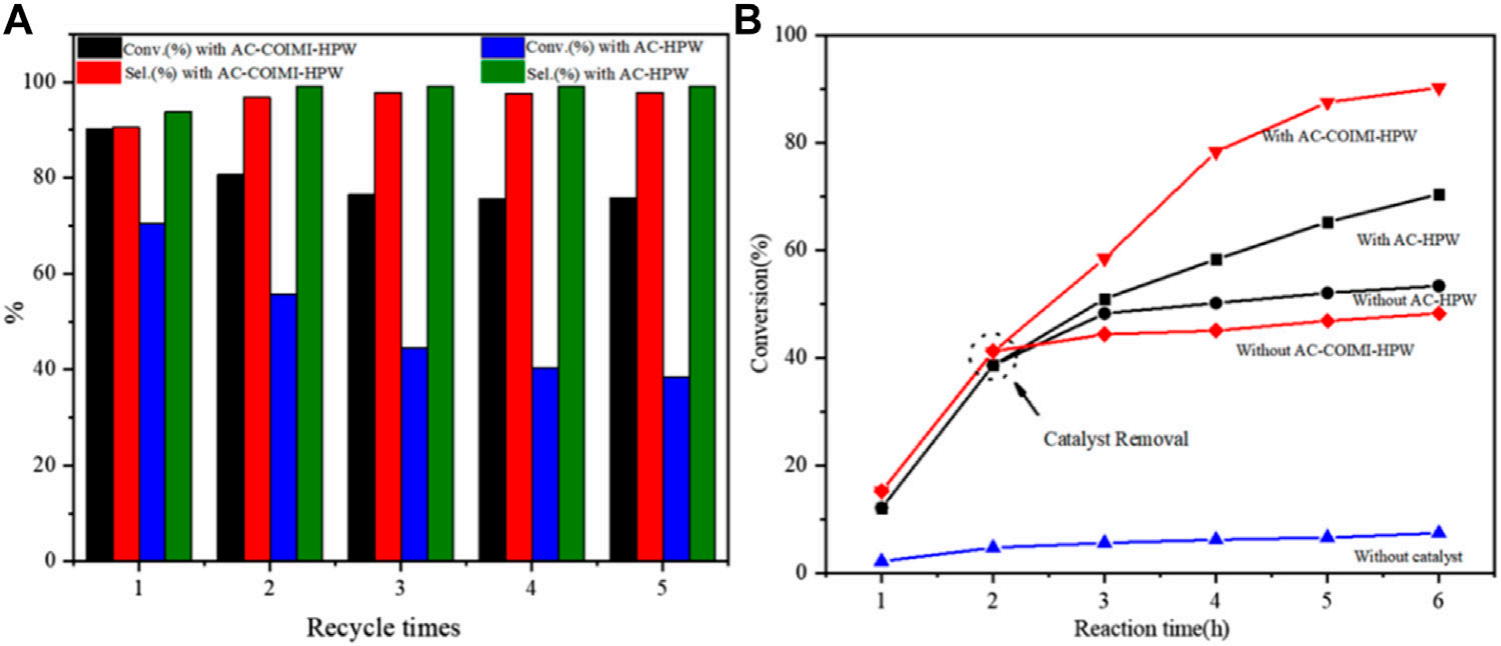
**(A)** Recycling experiment and **(B)** leaching experiment.

To understand the recyclability of AC-COIMI-HPW, the catalyst was usually separated, washed, and dried and then used for the next run. Technically, the drying operation is a consumption process for the recovery and is not suitable for industrial applications. In this work, the recycled catalyst was directly put into reuse in the next run, after centrifugal separation and transferring to the reaction flask with the solvent in the oxidation. Through the recycling experiment (Figure 7A), it could be seen that AC-COIMI-HPW could remain extremely stable when it was used three times. This indicated that the imidazole-modified activated carbon not only had higher catalytic activity than the unmodified activated carbon-supported phosphotungstic acid catalyst but also had better stability.

A leaching experiment was carried out in order to verify the decrease in the catalyst effect, and the results are displayed in Figure 7B. When the reaction reached 2 h, the reaction mixture in the flask was drawn out and centrifuged to separate the catalyst. The collected reaction liquid was allowed to react further without any catalyst. It could be seen that when the AC-COIMI-HPW catalyst was taken out, the conversion of benzyl alcohol was similar to that without a catalyst. Moreover, it could be found that the catalytic activity of AC-HPW was higher than that of AC-COIMI-HPW under the same reaction conditions. On the other hand, it is shown that benzyl alcohol conversion was slightly decreased, and the selectivity of benzaldehyde was elevated on both catalysts in the second run in Figure 7A. In order to clarify whether the leaching of phosphotungstic acid was responsible for the decrease in the catalytic activity, an ICP experiment was operated for the detection of the W element in the reaction mixture. As expected, it was found that a trace of phosphotungstic acid was dissolved in the first operation, and the detected amount from the reaction liquids on AC-HPW was more than on AC-COIMI-HPW (Table 5). It was observed that no W element was found in the AC-COIMI-HPW system after three recycling phases. This indicated that the activated carbon modified by imidazole anchored phosphotungstic acid through ionic bonds, which not only had better catalytic activity but also greatly improved its stability.

**TABLE 5:** ICP results of the W element for the samples of the reaction mixture after tests.

Catalyst	First^a^	Third^a^	Fifth^a^
AC-COIMI-HPW	0.017	Not detected	Not detected
AC-HPW	0.026	0.004	0.004

a W content in the reaction mixture (μg·ml^−1^).

In addition, it is clear seen that AC-COIMI-HPW worked as an oxidative catalyst, exhibiting excellent catalytic performance for the oxidation of benzyl alcohol to benzaldehyde as compared to other systems (Table 6).

**TABLE 6:** Comparison of the reported catalytic oxidation of benzyl alcohol with H_2_O_2_.

Catalyst	Temperature (°C)	Time (h)	Conversion (%)	Selectivity (%)	Times^a^	Reference
AC-COIMI-HPW	90	6	90.2	91.5	5	This work
[TMGHA]_2.4_H_0.6_PW	90	6	69.8	91.8	1	Chen et al. (2014)
GO–N–PW	100	6	80	95	3	Liu et al. (2014)
PAMAM dendritic phosphotungstate hybrids	100	6	89	91	5	Chen et al. (2014)
Long-chain multi-SO_3_H heteropolyanion	70	4	100	92	5	Li et al. (2015)
HPW/PEHA/ZrSBA-15	80	4	61.5	89.0	1	Zhang et al. (2015)
Polyoxometalate-based gemini ionic liquid	95	6	96	86	7	Hao et al. (2018)
Iron (III) tosylate	60	10	91.7	71.3	1	Zhao et al. (2018)
[PipBs_2_]_3_-(PW)_2_	90	3	96	93	5	Keshavarz et al. (2019)
Chromium borophosphate	80	8	58	95	1	Demet Ozer, (2020)
PW@IL-GO	100	5	93	91	5	Zheng et al. (2020)
Iron chloride–immobilized ionic liquid	90	6	61.8	73.6	7	Cang et al. (2020)

a The numeral referring to total times in recycling use of typical catalyst.

### 3.4 Adsorption and Catalytic Mechanism

**FIGURE 8:**
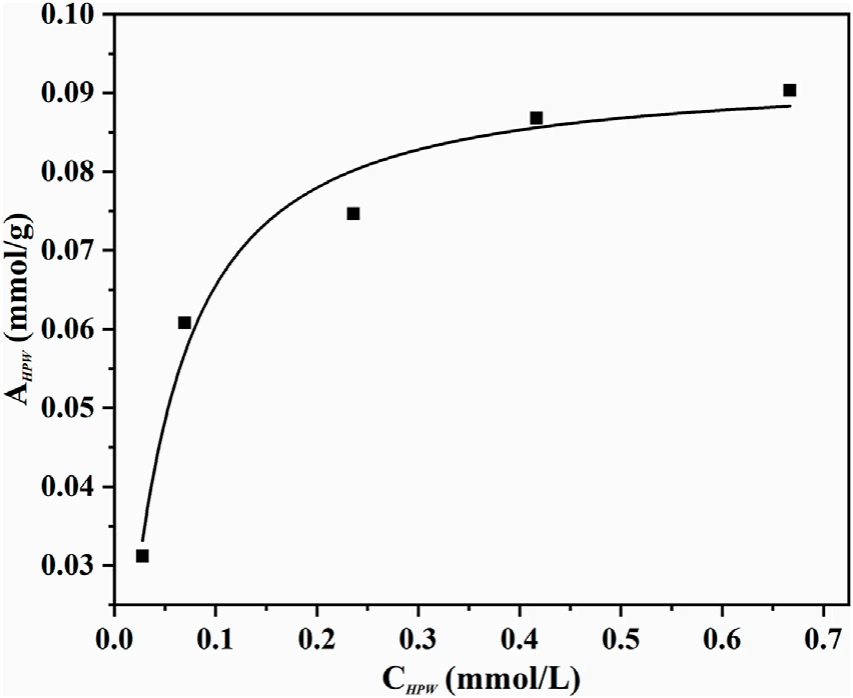
Adsorption isotherms of HPW on AC-COIMI (25°C). Langmuir isotherm equation could be expressed as follows.

An adsorption experiment was carried out for discriminating the distribution characteristics of phosphotungstic acid on the innovative catalyst AC-COIMI-HPW at 25°C. The AC-COIMI-HPW samples were added to a series of concentration gradient phosphotungstic acid solutions, and the actual HPW load relative to the equilibrium concentration was obtained by measuring the initial and final absorbance value with the UV-Vis spectrum. The adsorption isotherm is depicted in Figure 8. The Langmuir model or Freundlich adsorption model was used to compare the adaptability, respectively.

$$\frac{{\text{A}}_{HPW}}{{\text{A}}_{m,HPM}}=\frac{{\text{b}}_{HPW}{\text{C}}_{HPW}}{1+{\text{b}}_{HPW}{\text{C}}_{HPW}}.$$ (1)

In Eq. 1, C_*HPW*_ is the equilibrium concentration of HPW (mmol/L), A_*HPW*_ is the corresponding adsorption amount (mmol/g), A_m,__*HPW*_ is the maximal adsorption amount or saturated adsorption capacity (mmol/g) in the Langmuir adsorption model, and b_*HPW*_ is the Langmuir adsorption equilibrium constant or adsorption coefficient.

Equation 1 could easily be converted to Eq. 2:

$$\frac{1}{{\text{A}}_{HPW}}=\frac{1}{{\text{A}}_{m,HPW}}+\frac{1}{{\text{A}}_{m,HPW}{\text{b}}_{HPW}}\frac{1}{{\text{C}}_{HPW}}.$$ (2)

Freundlich isotherm equation could be expressed as Eq. 3:

$${\text{A}}_{HPW}={\text{K}}_{F}{\text{C}}_{HPW}^{\frac{1}{n}}.$$ (3)

Equation 4 could be obtained by taking the natural logarithm to Eq. 3.

$$\mathrm{In}{\text{A}}_{HPW}=\mathrm{In}{\text{K}}_{\text{F}}+\frac{1}{\text{n}}\mathrm{In}{\text{C}}_{\mathrm{HPW}}.$$ (4)

K_*F*_ and n, respectively, are the constants in the Freundlich adsorption model.

**FIGURE 9:**
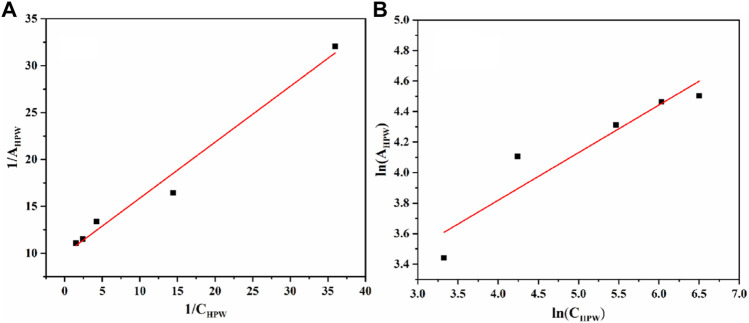
**(A)** Langmuir isotherm equation fitting. **(B)** Freundlich isotherm equation fitting.

As seen in Figure 9, it is found that the Langmuir adsorption model was more suitable, according to the comparison of the linear correlation of Eq. 2 (R^2^ = 0.975) with Eq. 4 (R^2^ = 0.851). The maximum adsorption capacity per unit mass of AC-COIMI-HPW, A_m, *HPW*_ = 97.6 μmol/g could be obtained. Surprisingly, the value of A_m, *HPW*_ was very consistent with the N-Base density of 103 μmol/g. It is revealed that HPW could be fixed on the surface of AC-COIMI by an acid-base ionic bond combination. Therefore, the catalytic stability of AC-COIMI-HPW was better than AC-HPW (Figure 7A).

In order to explain the differences in the catalytic activity of AC-COIMI-HPW (3), AC-COIMI, and HPW for benzyl alcohol conversion (Table 2, entries 2 and 3), a comparative experiment was performed, and the results are listed in Table 7. The catalytic results showed that at the same HPW loading, after the addition of 2-methylimidazole at 1:1 concentration, the catalytic performance was markedly modified for the oxidation of benzyl alcohol with H_2_O_2_. It should be noted that the effective catalytic species is 2-methyl imidazolium dihydrogen phosphotungstate, in which the acid strength of dihydrogen phosphotungstate anion is lower than phosphotungstic acid, so the redox potential of the catalytic species was adjusted to the synergetic activation of the substrates. In another consideration, the lipophilicity of 2-methyl imidazolium is beneficial for grasping benzyl alcohol and further sending the alcohol into the catalytic sphere of the dihydrogen phosphotungstate anion by π–π conjugation of a benzene ring and imidazole ring so that the activity and selectivity of AC-COIMI-HPW both improved the single phosphotungstic acid molecule adsorbed on the imidazole site of the AC-COIMI-activated carbon carrier.

**TABLE 7:** Effects of various catalysts on the oxidation reaction of benzyl alcohol.

Entry	2-Methylimidazole: HPW	Conversion (%)	Selectivity^a^ (%)
1	0:1	58.6	70.1
2	1:1	69.0	96.8

Reaction conditions: 4 mmol benzyl alcohol, 16 mmol H_2_O_2_, 0.075 mmol HPW t, 90°C, 6 h, and 15 ml solvent (CH_3_CN: H_2_O, 1: 3).

a Selectivity for benzaldehyde.

## 4 Conclusion

Keggin-type phosphotungstic acid was successfully hybridized with 2-methylimidazole-modified activated carbon through ionic interactions and hydrogen bonds, showing excellent characteristics of environmental friendliness, high efficiency, and easy reusability in the selective oxidation of alcohols using aqueous H_2_O_2_. The imidazole-functionalized activated carbon material in this work makes it not only easy to obtain the carrier from the activated carbon but also the modification method is also convenient for operation with aqueous H_2_O_2_ instead of nitric acid. It provides a practical innovative way for designing efficient heteropoly-based acid catalysts and possesses the potential for industrial application prospects.

## Data Availability

The original contributions presented in the study are included in the article/Supplementary Material; further inquiries can be directed to the corresponding author.
